# MALDI-imaging reveals thymosin beta-4 as an independent prognostic marker for colorectal cancer

**DOI:** 10.18632/oncotarget.6103

**Published:** 2015-11-05

**Authors:** Timo Gemoll, Sarah Strohkamp, Katharina Schillo, Christoph Thorns, Jens K. Habermann

**Affiliations:** ^1^ Section for Translational Surgical Oncology and Biobanking, Department of Surgery, University of Lübeck and University Medical Center Schleswig-Holstein, Campus Lübeck, Lübeck, Germany; ^2^ Department of Pathology, University Medical Center Schleswig-Holstein, Campus Lübeck, Lübeck, Germany

**Keywords:** mass spectrometry, genomic instability, aneuploidy, Tβ-4, prognosis

## Abstract

DNA aneuploidy has been identified as a prognostic factor for epithelial malignancies. Matrix-assisted laser desorption/ionization (MALDI) imaging mass spectrometry (IMS) is a powerful tool for direct analysis of multiple proteins in tissue sections while maintaining the cellular and molecular integrity. We compared diploid and aneuploid colon cancer tissues against normal mucosa of the colon by means of IMS.

DNA image cytometry determined the ploidy status of tissue samples that were subsequently subjected to MALDI-IMS. After obtaining protein profiles through direct analysis of tissue sections, a discovery and independent validation set were used to predict ploidy status by applying proteomic classification algorithms [Supervised Neural Network (SNN) and Receiver Operating Characteristic (ROC)]. Five peaks (m/z 2,395 and 4,977 for *diploid vs. aneuploid comparison* as well as m/z 3,376, 6,663, and 8,581 for *normal mucosa vs. carcinoma comparison*) were significant in both SNN and ROC analysis. Among these, m/z 4,977 was identified as thymosin beta 4 (Tβ-4). Tβ-4 was subsequently validated in clinical samples using a tissue microarray to predict overall survival in colon cancer patients.

## INTRODUCTION

Colorectal carcinomas (CRC) constitute the third most common malignancy in the Western World. Although numerous molecular events are well discussed during the development of colorectal cancer, predicting outcome and response to therapy still remains a major challenge for individualized medicine. Two main molecular subtypes of CRC are known that are associated with distinct clinical prognosis: chromosomal instability (CIN) and microsatellite instability (MSI). About 15–20% of sporadic cancers develop according to the MSI pathway and are characterized by a loss of the DNA mismatch repair (MMR) system. By failing to repair spontaneous errors that occur during replication, these tumors accumulate frame-shift mutations affecting tumor suppressor genes [1]. MSI tumors are known to present diploid tumor cell populations with few, karyotypic abnormalities, respond differentially to fluorouracil-based chemotherapy and show a favorable clinical prognosis [2, 3]. Microsatellite instability testing in clinical routine is performed on tumor tissue by means of PCR, immunohistochemistry (IHC) and MLH1 promoter methylation detection when patients fulfill the Bethesda guidelines and are suspected to have a Hereditary Non-Polyposis Colorectal Cancer Syndrome (HNPCC or Lynch-Syndrome) [4]. If test results are positive for IHC and/or MSI, molecular genetic testing is recommended in order to allow predictive testing for healthy family members.

In contrast, about 80% of CRCs show CIN, reflected by abnormal and highly scattered DNA stem line values. CIN cancers are associated with disease recurrence, metastases, and an inferior prognosis [5, 6]. Among routine prognosis markers, aneuploidy even proved to be the strongest independent prognostic marker in 260 R0 resected CRC patients [7]. Additionally, distinct ploidy-associated protein expression patterns were detected in colorectal cell lines that were also validated clinically using immunohistochemistry on a tissue microarray compromised of 31 diploid and 47 aneuploid CRCs [8]. However, acquiring knowledge of the underlying molecular mechanisms characterized by chromosomal abnormalities remains a major challenge for colorectal cancer development. CIN testing is performed via *APC* gene mutation analysis if the clinical diagnosis suggests a Familiar Adenomatosis Polyposis (FAP) Syndrome [9].

One drawback of biomarker screening technologies is the missing intra-tumoral spatial information due to the analysis of entire tissue sections with the compilation of multiple cells of different types (e.g. epithelial cancer cells, mucosa cells, muscle cells). In order to solve this problem, matrix-assisted laser desorption/ionization (MALDI) imaging mass spectrometry (IMS) has matured recently and provides a powerful tool for investigating proteins through the direct *in situ* analysis of thin tissue sections without time consuming preparation steps such as laser capture microdissection (LCM) [10, 11]. With the direct correlation of molecular information with traditional histology by keeping the spatial resolution, IMS enables measurement of both the distribution and the relative abundance of large biomolecules [12, 13], lipids [14, 15] and small molecules [16, 17] down to a near-cellular resolution level [18, 19]. A major advantage of IMS is the label-free annotation of tissues based on MS profiles and thereby the separation of distinct histological tissue regions without applying target-specific reagents and methods [20, 21]. Due to the heterogeneity of clinical tissue samples, IMS has therefore the great potential to enable subclassification for individualized medicine in terms of diagnostic, therapeutic and prognostification means.

Against this background, we have now studied whether an aneuploidy-associated protein expression signature is detectable by MALDI-IMS in clinical tissues. An identified target was further tested for its prognostic value.

## RESULTS

### Ploidy assessment

DNA image cytometry classified three normal mucosa samples, three diploid and three aneuploid colon carcinomas to be analysed in this study (Table 1a; Supplemental data 1). One aneuploid tumor sample showed no invasive carcinoma during histopathological re-evaluation on consecutive slides and was thus excluded from further experiments.

**Table 1A:** Clinical characteristics of tissue samples used for IMS

		Clinical data							Ploidy measurement			
#	Group type	Sex	Age	T	N	M	G	UICC	Classification	Cells	SL	5c-Exc
T1	tumor	w	70	2	0	0	3	1	diploid	1,598	2.02	1
T2	tumor	m	71	3	0	0	2	2	diploid	8,284	1.96	2
N2	normal mucosa								diploid			
T3	tumor	m	66	4	2	1	3	4	diploid	4,791	1.97	2
N3	normal mucosa								diploid			
T4	tumor	m*	69	2	0	0	3	1	aneuploid	1,083	2.01 + 5.67	162
T5	tumor	w	64	3	1	0	2	3	aneuploid	3,621	2.03	30
T6	tumor	w	62	4	1	1	2	4	aneuploid	1,912	1.95 + 3.27	142
N6	normal mucosa								diploid			

SL, stem lines; 5c-Exc, Number of measured cells with a DNA content over 5c

* excluded from MALDI-IMS experiments due to missing invasive carcinoma on consecutive slides after histopathological re-evaluation

**Table 1B:** Clinical characteristics of colon cancer patients for tissue microarray (TMA) analysis with measured immunopositivity by immunohistochemistry

Sex	Age	Type	Ploidy	T	N	M	G	UICC	Survival [Months]	Status [Dead/alive]	IP
m	59	normal	mucosa								0.1022
m	58	normal	mucosa								0.0881
w	78	normal	mucosa								0.0052
m	57	normal	mucosa								0.0459
w	52	normal	mucosa								0.0018
m	74	normal	mucosa								0.0099
m	47	normal	mucosa								0.1199
w	51	normal	mucosa								0.0330
m	66	normal	mucosa								0.0038
w	74	normal	mucosa								0.1991
m	59	tumor	diploid	3	0	0	2	1	90.0	alive	0.0020
w	61	tumor	diploid	4	0	0	2	1	154.0	alive	0.2673
w	75	tumor	diploid	3	0	0	2	1	57.6	dead	0.2191
m	58	tumor	diploid	3	0	0	2	1	40.8	dead	0.5336
w	78	tumor	diploid	4	0	0	2	1	50.4	dead	0.4410
m	77	tumor	diploid	2	1	0	3	2	114.0	alive	0.2467
m	57	tumor	diploid	3	1	0	2	2	64.8	alive	0.4448
w	52	tumor	diploid	2	1	0	2	2	142	alive	0.2371
w	73	tumor	diploid	4	1	0	3	2	18.0	dead	0.2715
m	74	tumor	aneuploid	3	0	0	2	1	142.0	alive	0.3007
m	47	tumor	aneuploid	3	0	0	3	1	133.0	alive	0.1592
w	74	tumor	aneuploid	3	0	0	2	1	176.0	alive	0.3615
w	72	tumor	aneuploid	3	0	0	2	1	27.6	dead	0.7899
m	64	tumor	aneuploid	3	0	0	3	1	55.2	dead	0.4529
w	51	tumor	aneuploid	3	1	0	2	2	109.0	alive	0.2003
m	66	tumor	aneuploid	4	1	0	3	2	145.0	alive	0.0780
w	74	tumor	aneuploid	3	1	0	3	2	10.8	dead	0.5090
w	63	tumor	aneuploid	3	1	0	2	2	27.6	dead	0.6380

IP, immunopositivity

### MALDI imaging and statistical evaluation

MALDI imaging was applied to five colon carcinomas, representing different ploidy patterns, and to three normal mucosa tissues. On every section, the regions of interest (ROI) were defined, resembling representative regions of predominantly cancerous and non-cancerous areas, respectively. Within the mass range from m/z 2 to 25, differentially expressed protein peaks between samples of normal mucosa and colon cancer as well as between diploid and aneuploid colon cancer were detected. Overall, individual and sum spectra of ROI are demonstrated in Supplemental data 2 and 3.

**Figure 1:**
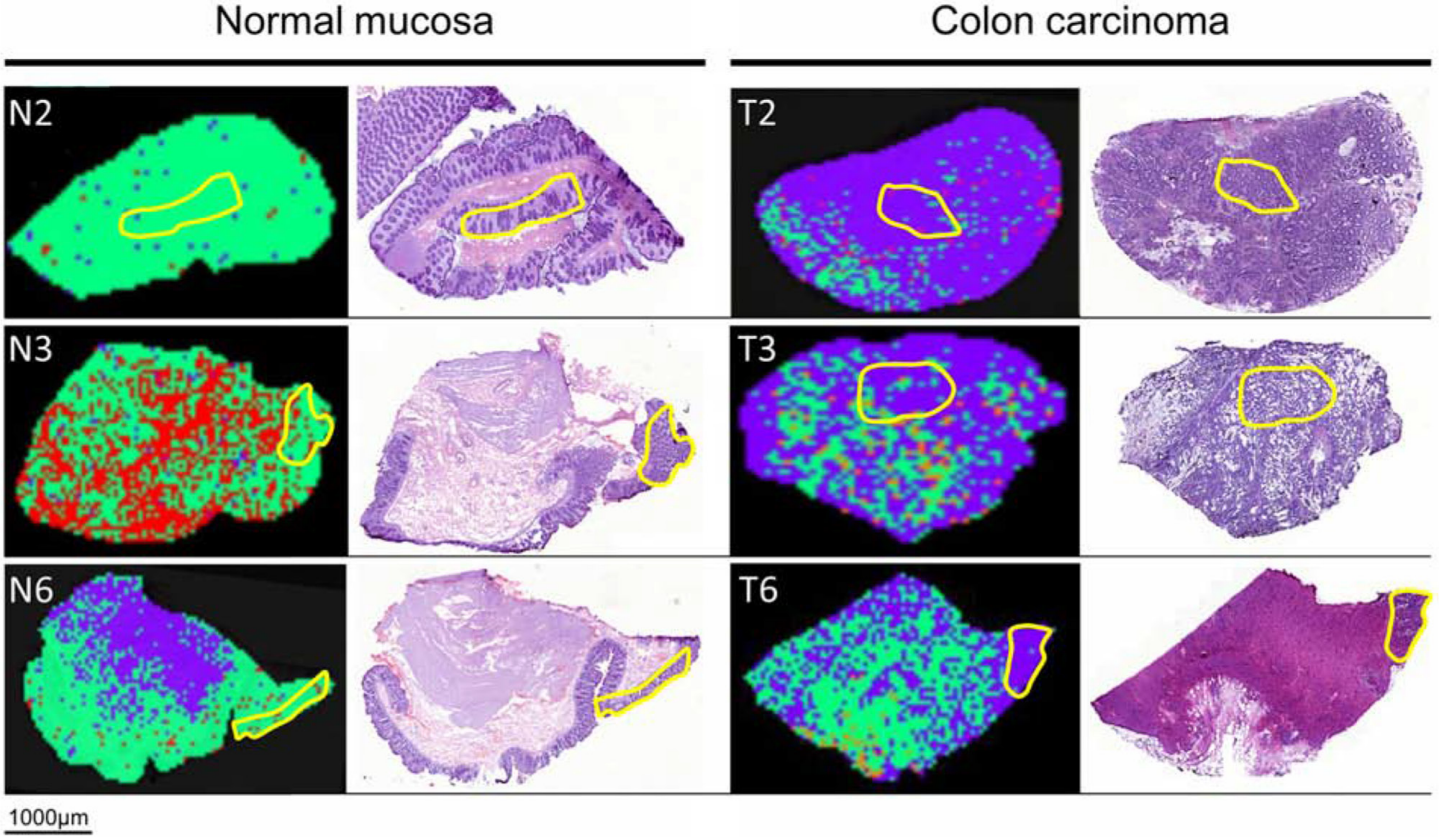
MALDI-IMS images and H&E staining of the same section of adjacent normal mucosa (left) and adjacent colon carcinoma (right) Yellow outlines represent pathological regions of interests (ROI) to detect m/z values of adjacent normal mucosa and adjacent colon carcinoma for generating the Supervised Neural Network (SNN) algorithm. Applying the SNN algorithm of 15 peaks to the entire slides (validation set) enables discrimination between carcinoma and adjacent normal mucosa. Green dots are pixels assigned by the SNN model to indicate adjacent normal mucosa; purple dots are pixels assigned to indicate carcinoma tissue. Red areas remained unclassified.

**Figure 2:**
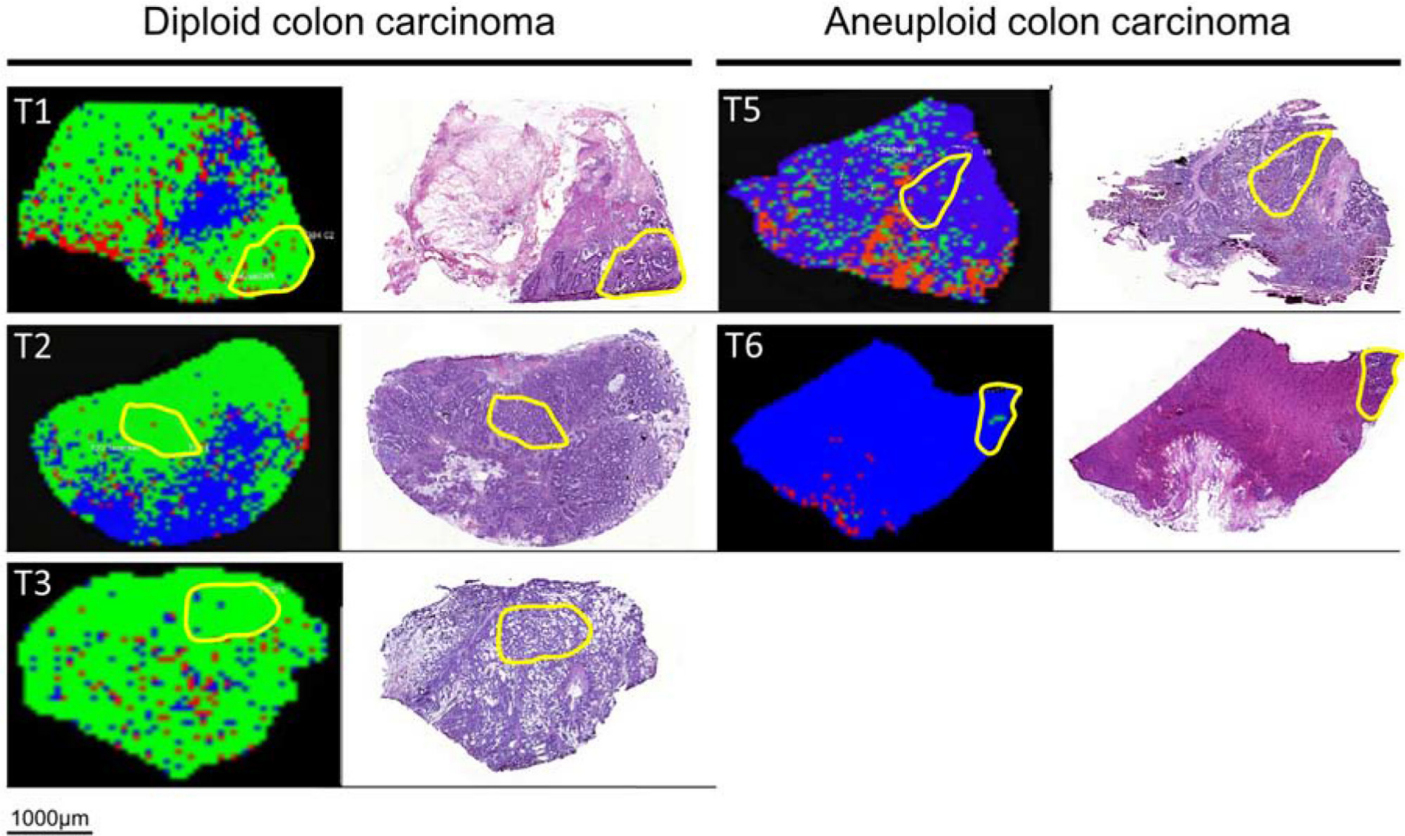
MALDI-IMS images and H&E staining of the same section of diploid (left) and aneuploid colon carcinoma (right) Yellow outlines represent pathological regions of interests (ROI) to detect m/z values of diploid and aneuploid colon carcinomas for generating the Supervised Neural Network (SNN) algorithm. Applying the SNN algorithm of 12 peaks to the entire slides (validation set) enables discrimination between diploid and aneuploid colon cancers. Green dots are pixels assigned by the SNN model to indicate diploid carcinoma cell populations; blue dots are pixels assigned to aneuploid carcinoma cell populations. Red areas remained unclassified.

Data analysis was performed using ClinProTools software 2.2 (Bruker Daltonic GmbH, Germany) to distinguish between groups. Hereby, a discovery set was computed to predict ploidy and disease status by applying proteomic classification algorithms [Supervised Neural Network (SNN), Receiver Operating Characteristic (ROC)]. The generated models were used to classify each spectrum of samples into distinct groups. Based on a SNN algorithm, 90% of the colon carcinomas and 99% of the normal mucosa samples were categorized correctly. This algorithm was based on 15 peaks of different masses ranging from m/z 2,400–22,700 (Table 2). Accordingly, 99% of diploid and 94% of aneuploid colon cancers were classified correctly by using the SNN algorithm for 12 peaks in a range from m/z 2,400–15,700 (Table 3). Subsequent validation of both comparisons in new ROIs showed a correct classification of 92% diploid and 78% aneuploid colon cancers and 92% of normal mucosa and 96% of tumors, respectively. Additionally, the classification of the entire slides was successful for the *normal mucosa vs. carcinoma comparison* as well as for the *diploid vs. aneuploid comparison* (Figures 1 & 2).

**Table 2:** Peak list of the SNN-based model for the normal mucosa vs. carcinoma comparison

IMS protein mass [m/z]	Weight	AUC > 0.75	Protein names* (Short name^+^, Uniprot-ID)	Protein mass* [m/z range]
2,434	0.0212		Stress-70 protein, mitochondrial (mtHSP70, GRP_Human) Protein disulfide-isomerase (PDI, PDIA1_Human) Desmoplakin (DP, DESP_Human) Exportin-2 (Exp2, XPO2_Human) Translocator protein (TSPO, TSPOA_Human) Mucin-2 (MUC-2, MUC2_Human) Voltage-dependent anion-selective channel protein 1 (VDAC-1, VDAC1_Human) Glutamine-fructose-6-phosphate amidotransferase 1 (GFAT1, GFPT1_Human) Eukaryotic translation initiation factor 2 subunit alpha (eIF-2A, IF2A_Human) Heterogeneous nuclear ribonucleoproteins A2/B1 (hnRNP A2/B1, ROA2_Human) Transketolase (TK, TKT_Human) Protein S100-A1 (S10A1_Human) Adipocyte enhancer-binding protein 1 (AE-binding protein 1, AEBP1_Human) Ezrin (EZRI_Human)	2,433.01 – 2,434.25
3,008	0.0489		Ras GTPase-activating-like protein IQGAP1 (IQGA1_Human) Glutathione S-transferase P (GSTP1–1, GSTP1_Human) 2′, 3′-cyclic-nucleotide 3′-phosphodiesterase (CNPase, CN37_Human) Collagen alpha-1(XII) chain (COCA1_Human)	3,007.48 – 3,008.60
3,334	0.0247		Major vault protein (MVP, MVP_Human) Ig gamma-1 chain C region (IGHG1_Human) IGHV4–31 protein (A0A087WSY4_Human)	3,334.65 – 3,334.74
3,376	0.0580	0.80	14–3-3 protein sigma (14–3-3(, 1433S_Human)	3,376.60
3,448	0.0760		Tubulin alpha-1C chain (Q8N532_Human) Collagen alpha-1(VI) chain (CO6A1_Human) Tubulin alpha-1B chain (TBA1B_Human)	3,448.64 – 3,448.66
5,150	0.0347			
6,203	0.0270			
6,663	0.0501	0.86		
7,017	0.0381			
8,581	0.0763	0.88		
9,274	0.0375			
20,804	0.0415			
22,238	0.0334			
22,533	0.0338			
22,738	0.0392			

IMS, MALDI-imaging

* identified by Maier et al. within the deviation of m/z ±1 [25]

+ if specified at http://www.uniprot.org

**Table 3:** Peak list of the SNN-based model for the diploid vs. aneuploid comparison

IMS protein mass [m/z]	Weight	AUC > 0.75	Protein names* (Short name^+^, Uniprot-ID)	Protein mass* [m/z range]
2,395	0.1311	0.80	Eosinophil peroxidase (EPO, PERE_Human) Tubulin beta-4B chain (TBB4B_Human) Epiplakin (EPIPL_Human) Cullin-3 (CUL-3, CUL3_Human) Prolow-density lipoprotein receptor-related protein 1 (LRP-1, LRP1_Human) Extended synaptotagmin-1 (E-Syt1, ESYT1_Human) Transgelin (TAGL_Human) DNA-dependent protein kinase catalytic subunit (DNA-PKcs, PRKDC_Human) Peroxisomal acyl-coenzyme A oxidase 1 (AOX, ACOX1_Human) Protein deglycase DJ-1 (DJ-1, PARK7_Human) Lactotransferrin (TRFL_Human), Protein enabled homolog (ENAH_Human) Transgelin-2 (TAGL2_Human) Tubulin protein (Q9BUU9_Human)	2,394.05 – 2,395.32
4,761	0.0693		Thymosin beta-4 (TΔ-4, TYB4_Human)	4,761.42
4,798	0.0553			
4,977	0.0667	0.81	Thymosin beta-4 (TΔ-4, TYB4_Human)	4,977.49
6,501	0.0249			
6,585	0.1204			
6,663	0.0774			
7,765	0.0358			
7,801	0.0273			
8,787	0.0537			
12,297	0.0245			
15,719	0.0298			

IMS, MALDI-imaging

* identified by Maier et al. within the deviation of m/z ± 1 [25]

+ if specified at http://www.uniprot.org

For ROC analysis, 12 peaks in the *diploid vs. aneuploid comparison* and 4 peaks in the *normal mucosa vs. carcinoma comparison* showed an AUC >0.75. Interestingly, five peaks (m/z 3,376, 6,663, and 8,581 for the *normal mucosa vs. carcinoma comparison* as well as m/z 2,395 and 4,977 for the *diploid vs. aneuploid comparison*) were observed to be significant in both, SNN and ROC analysis and strongly correlated with colon cancer morphology and aneuploidy (Table 2 & 3).

### Identification and histological validation of thymosin beta-4 (Tβ-4)

For identification, differential mass peaks (*n* = 8) were analysed by *MaTisse* database search. The complete bottom-up matrix and tissue proteomes as well as the top-down identifications in *MaTisse* revealed in total 40 different proteins for the eight significant m/z-values. Thymosin β-4 (Tβ-4) and 14-3-3 protein sigma (14-3-3-σ) were the only proteins showing a single identification hit of which Tβ-4 was detected to be differently regulated between diploid and aneuploid colon cancers (Tables 2 & 3; Supplemental data 4 & 5).

**Figure 3:**
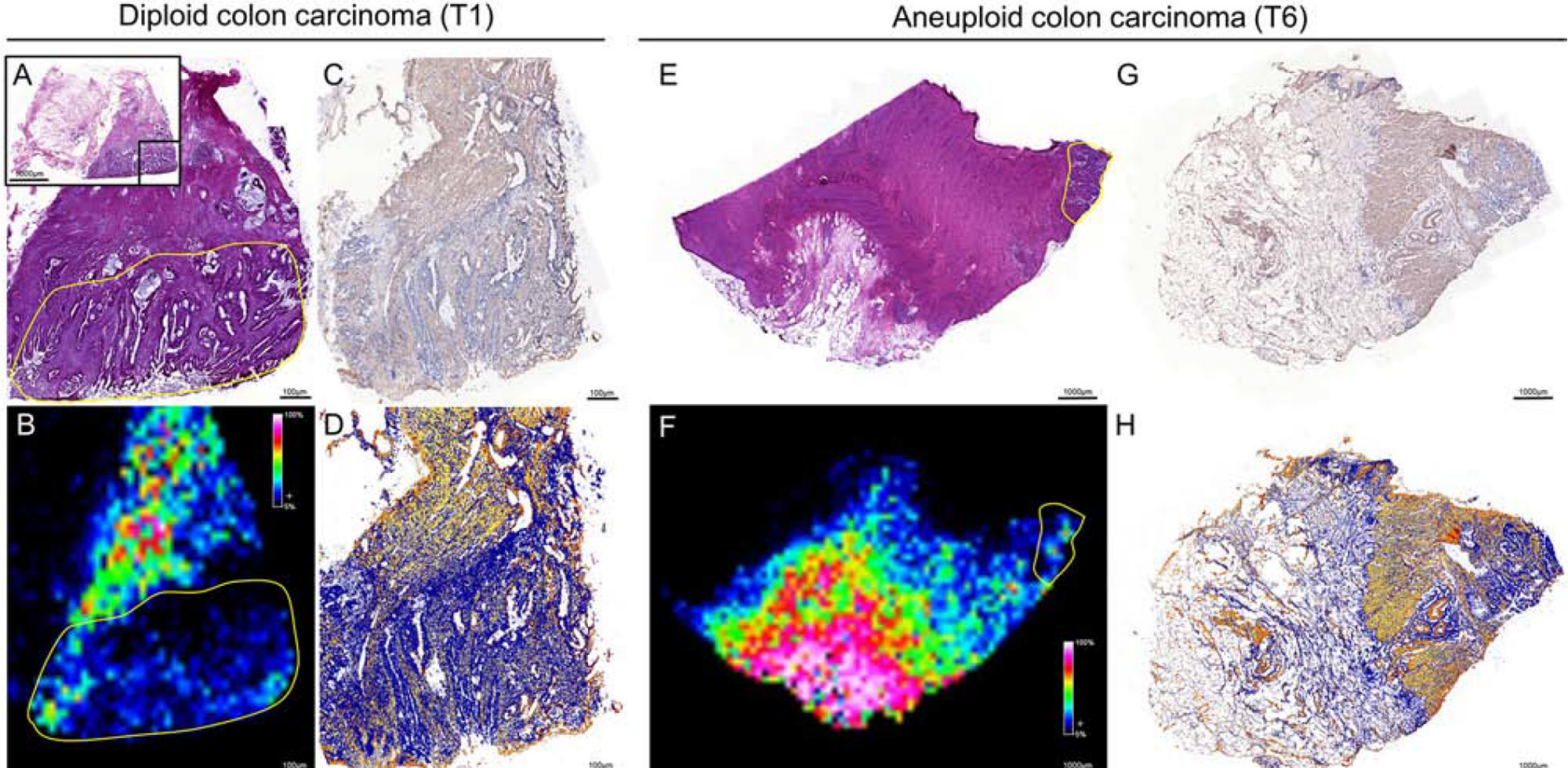
Validation of the histological distribution of Tβ-4 by immunohistochemistry in diploid (left) and aneuploid (right) colon carcinomas With respect to histology of HE stained tissue **A & E,** a close correlation between the IMS signal of Tβ-4 (m/z value 4,977) **B & F** and the immunopositivity of the antibody against Tβ-4 **C & G** was observed. Immunopositivity of the antibody against Tβ-4 was evaluated by ImageScope **D & H.** Yellow outlines represent regions of interests (ROI) on the basis of pathological classification in order to select m/z values of diploid and aneuploid cancer cell populations.

Subsequently, quantity and spatial distribution of Tβ-4 in colon cancer were validated by IHC. Hereby, a consecutive slide of a diploid and aneuploid fresh frozen carcinoma was used that could be correlated to the same malignant and non-malignant tissue histology measured by IMS. By comparing the IMS distribution of Tβ-4 (m/z 4,977) and the immunopositivity (IP) of Tβ-4 immunohistochemical staining, a close correlation of the Tβ-4 intensity with respect to histology was observed (Figure 3).

**Figure 4:**
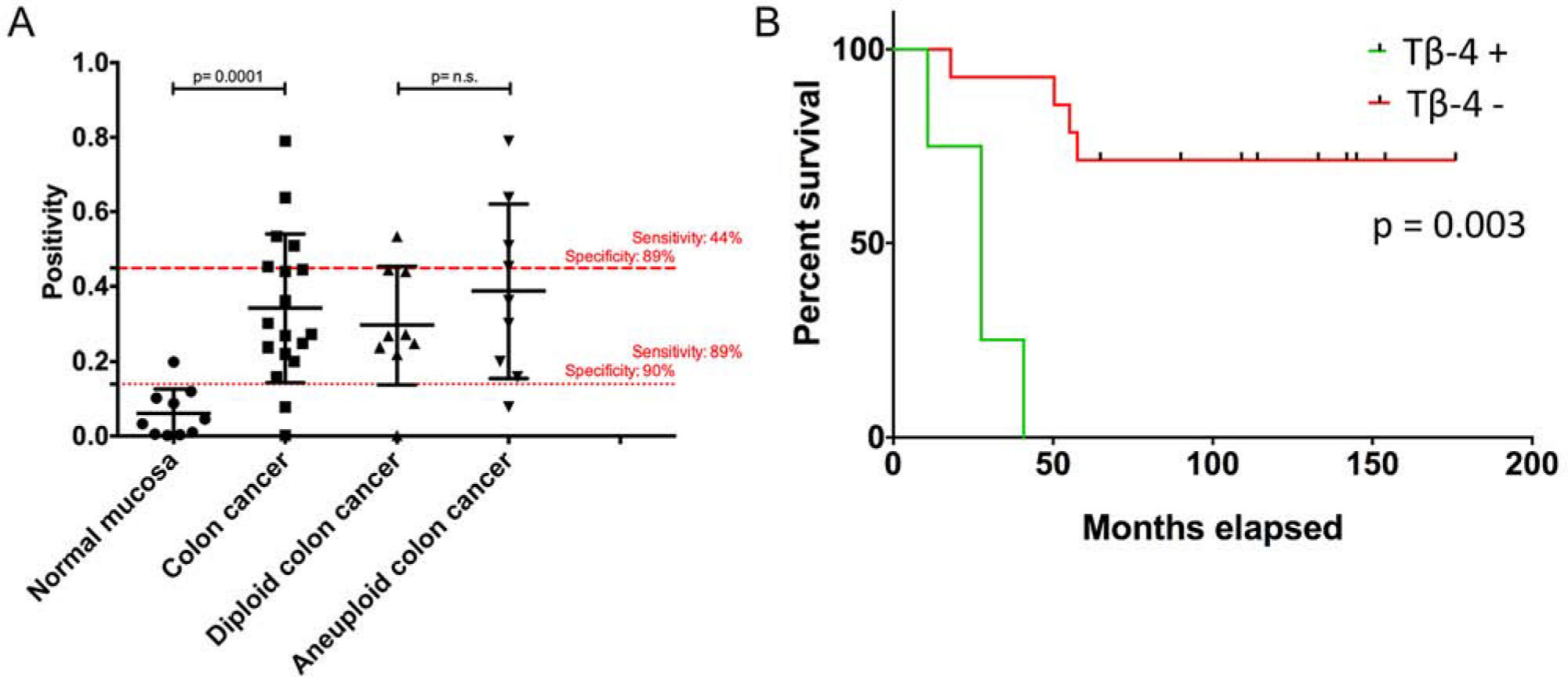
(A) Tissue-microarray-based immunohistochemical evaluation of Tβ-4 by means of Image scope comparing normal mucosa vs. colorectal carcinomas and diploid versus aneuploid colorectal carcinomas Red lines represent cut-off values for the normal mucosa vs. CRC comparison (^…^) and for the diploid vs. aneuploid CRC comparison (- - -) with highest sensitivity and specificity. **B.** Overall survival curves of colorectal carcinoma patients depending on the occurrence of Tβ-4 positivity. Comparison of patients without (Tβ-4-) and with (Tβ-4+) Tβ-4 immunohistochemical positivity throughout a 174-months interval after dichotomization of samples based on the highest sensitivity and specificity between diploid and aneuploid CRCs (IP cut-off: 0.4489 with sensitivity of 44% at 89% specificity).

Subsequently, clinical relevance was shown by tissue microarray (TMA)-based immunohistochemistry of matched clinical samples (*n* = 28). Tβ-4 cytoplasmatic immunopositivity was more frequently present in aneuploid (median = 0.3615) than in diploid (median = 0.2673) carcinomas. Although this difference did not reach significance, Tβ-4 immunohistochemistry showed a similar trend as in the IMS analysis with an up-regulation in aneuploid samples. However, after dichotomization of samples based on the highest sensitivity and specificity between diploid and aneuploid colon cancers into Tβ-4- and Tβ-4+ groups (IP cut-off: 0.4489 with sensitivity of 44% at 89% specificity; Figure 4a), Tβ-4 proved as a predictive parameter for overall survival (HR, 12.959; 95% CI, 2.352 – 71.399; *p* = 0.003). Thus, overall survival was strikingly impaired with expression of Tβ-4 (Tβ-4+; Figure 4b). The binary regression between ploidy status and Tβ-4 expression was not significant.

Interestingly and although not detected by means of IMS, Tβ-4 showed a high significant expression difference between normal adjacent mucosa (median = 0.0395) and all carcinoma specimens (median = 0.2861) irrespective of their ploidy status (*p* = 0.0001, Figure 4a) with a sensitivity of 89% at 100% specificity.

## DISCUSSION

The ongoing search for new and reliable tumor markers for individualized medicine of colorectal cancer is underlined by the fact that it has become one of the most commonly diagnosed malignancies worldwide [22]. Identifying biomarkers could improve patients' prognosis and treatment. The results presented in this paper demonstrate that MALDI-IMS analysis of diploid and aneuploid colorectal cancer tissue detects an altered intensity in a number of characteristic m/z peaks which, when analysed by SNNs and ROCs, show a correct classification of each group in over 78% of the cases. By applying this algorithm to every spectrum of a section obtained by MALDI-IMS, diploid and aneuploid subgroups could be distinguished (Figure 2). The different classification rates for diploid (92%) and aneuploid (78%) tumors could be explained by the varying grade of genomic instability within aneuploid tumors, e.g. with multiple stem lines ranging from 2c to 5c (Table 1a) or even beyond. Thus, inter-tumor heterogeneity of aneuploid tumors is expected to be higher as in diploid tumors. Additionally, the varying degree of genomic instability can also cause increased intra-tumor heterogeneity that in turn makes correct classification even more difficult in aneuploid than in diploid tumors. Nevertheless, our MALDI-IMS results were subsequently validated and predict overall survival in colorectal cancer patients.

Interestingly and despite differences in (pre-)analytical techniques, two m/z values with differential intensities between diploid and aneuploid tumor samples were detected (m/z 6,663 and m/z 3,376) that have been published previously [23, 24]. Whereas Meding et al. identified the m/z value 6,663 as a marker for regional lymph node metastasis in colorectal cancer, the group of Alexandrov and colleagues detect m/z 3,376 as one of 23 co-localized m/z values that represents dissociated growing tumor populations of human larynx carcinoma.

Next to m/z 6,663 and m/z 3,376, our segmentation approach elucidated one highly intense m/z signal (m/z 4,977) distinctively associated with aneuploidy. While MALDI-imaging offers in fact the possibility to directly obtain the protein identity of m/z peaks by on-tissue trypsination and tandem-MS, a more classical top-down approach is electrospray MS. Here, the corresponding masses could be assigned to thymosin β-4 (Tβ-4) by Maier et al. and using the *MaTisse* database [25]. Furthermore, Tβ-4 was identified by Schey et al. who used an LC-MS/MS approach coupled with electron transfer dissociation (ETD) [26]. The corresponding m/z value with respect to its histological location was subsequently validated by immunohistochemistry (Figure 3).

Tβ-4 is an actin-sequestering molecule and is thus primarily associated with structural activities of the cytoskeleton and with basic physiologic functions like differentiation, wound healing, cell motility and the inflammatory response [27–29]. Tβ-4 is found in the nucleus as well as in the cytoplasma. There is compelling evidence that Tβ-4 plays a major role in facilitating tumor metastasis and angiogenesis [27, 30]. In this context, Tβ-4 expression is elevated in metastatic melanoma cells and breast cancer cells [31, 32]. In line, fibrosarcoma cells possessing greater metastatic potential express high levels of Tβ-4, whereas fibrosarcoma cells of low metastatic potential express little or no thymosin β4 [33]. For CRC, upregulation of the *Tβ-4* gene was found to correlate with increased invasion of colon carcinoma cells as well as liver metastasis in CRC patients [29]. As it has been also shown that an increased Tβ-4 expression induces invasion of human colorectal cancer cells through epithelial-to-mesenchymal transition (EMT) pathways [34], critical steps during cancer metastasis seem to be modulated by Tβ-4.

Additionally, it has been shown that Tβ-4 may drive the development of colorectal adenocarcinoma during multistage carcinogenesis [35]. TMA-based validation in this study confirmed these findings: while Tβ-4 immunoreactivity was absent or weak in normal mucosa, carcinomas showed moderate to strong immunoreactivity (*p* = 0.0001). Furthermore, a more frequent Tβ-4 immunopositivity characterizes aneuploid carcinomas that are known to show an inferior prognosis than diploid carcinomas [6]. Based on the highest sensitivity and specificity between diploid and aneuploid colorectal cancers, it could be additionally shown that increased Tβ-4 protein level was associated with reduced survival of patients with colorectal cancer (Figure 4b).

In conclusion, our data show that using small amounts of fresh-frozen tumor tissue easily available in cooperation with a hospital integrated biobank, protein profiles obtained from unprocessed tissue samples can be used to accurately classify colon tumors from normal mucosa. Beyond classification of tumor status, MALDI-IMS also allows discerning accurately between genomically stable (diploid) and instable (aneuploid) tumor types reflecting different biology and prognosis. On that basis, Tβ-4 was identified and validated as a CRC-associated protein with close correlation to genomic instability. Remarkably, Tβ-4 was further associated with poor prognosis and could potentially serve as a molecular target for tailored anticancer therapy.

## MATERIALS AND METHODS

### Sample preparation

For MALDI imaging experiments, colon carcinomas (*n* = 6) and adjacent normal mucosa (*n* = 3) were analyzed (Table 1a). For immunohistochemical validation, tissues of ten normal mucosa and 20 colorectal carcinomas were implemented into an in-house compiled tissue microarray (Table 1b). Clinical material was collected from surgically removed tissue adhering to guidelines of the local ethical review board (# 07–124). Prior to storage in liquid nitrogen, carcinoma tissue was used for touch preparation slides (imprints) for nuclear DNA content measurements by image cytometry. In addition, paraffin-embedded specimens of all samples were used for hematoxylin-eosin (HE) stained sections in routine histopathology.

### Genomic instability assessment

Genomic instability was assessed by nuclear DNA ploidy measurements by means of image cytometry using Feulgen stained imprints. The staining procedure, internal standardization, and cell selection criteria have been described previously [36]. Briefly, at least 500 nuclei per imprint were selected interactively and the DNA content was measured quantitatively using the ACAS imaging system (Ahrens ACAS, Hamburg, Germany). All DNA values were expressed in relation to the corresponding staining controls (lymphocytes), which were given the value 2c, denoting the normal diploid DNA content. The DNA profiles were classified according to Auer (Supplemental data 6) [36].

Histograms characterized by a single peak in the diploid or near-diploid region (1.5 – 2.5c) were classified as type I. The total number of cells with DNA values exceeding the diploid region (>2.5c) was <10%. Type II histograms showed a single peak in the tetraploid region (3.5 – 4.5c) or peaks in both the diploid and tetraploid regions (>90% of the total cell population). The number of cells with DNA values between the diploid and tetraploid region and those exceeding the tetraploid region (>4.5c) was <10%. Type III histograms represented highly proliferating near-diploid cell populations and were characterized by DNA values ranging between the diploid and the tetraploid region. Only a few cells (<5%) showed more than 4.5c. DNA histograms of types I, II, and III thus characterize euploid cell populations. Type IV histograms showed increased (>5%) and / or distinctly scattered DNA values exceeding the tetraploid region (>4.5c) reflecting aneuploid populations of colon mucosa nuclei with decreased genomic stability. All DNA histograms were evaluated by three independent investigators who were unaware of the clinical and histopathological data of the patients.

### MALDI-IMS experiments

Tumor and normal mucosa frozen tissues (*n* = 9) were sectioned at 10 μm thickness by a cryostat (Leica Microsystem, Germany) and then thaw-mounted onto an ITO-coated glass slide (Bruker Daltonics, Germany). After brief washes in 70% and 100% ethanol, sections were dried under vacuum. Spray-coating of the sections with matrix solution [20 mg/ml sinapinic acid (Sigma Aldrich, USA) in water/acetonitrile 50:50 (v/v) with 0.2% trifluoroacetic acid (Sigma Aldrich, USA)] was subsequently performed manually using a pneumatic Thin Layer Chromatography (TLC) sprayer (Sigma Aldrich, USA) with a constant nitrogen flow. In order to test reproducibility of matrix coating, the homogeneity of crystallized matrix layer was verified microscopically after 20 spray cycles. MALDI Imaging analyses were performed on an Ultraflex II time-of-flight mass spectrometer (Bruker Daltonics, Germany) with a SmartBeam laser operating at 200Hz in positive linear mode using FlexControl 3.0 and FlexImaging 2.1 software packages (Bruker Daltonics, Germany). Ions were detected in the 2,000–25,000 *m/z* mass range with a sampling rate of 0.1 GS/s. The lateral resolution for MALDI-IMS was set to 90 μm and a total of 200 laser shots were accumulated per pixel at constant laser power. A standard protein mixture (Bruker Daltonics, Germany) was employed for spectra calibration, which was done externally on the same target before each measurement. A section, adjacent to the analyzed one, was stained with hematoxylin and eosin, scanned and co-registered with the MALDI-IMS image in order to correlate mass spectrometric data with the histological features of the same section.

### Identification and histological validation of Tβ-4 by immunohistochemistry

After IMS, protein identification was carried out by mining the *MaTisse* database search (www.wzw.tum.de/bioanalytik/matisse). *MaTisse* is a publically available database consisting of the analysis of ten human tissues by IMS that lead to the identification of 1,400 abundant and soluble proteins [25]. Search parameters included a mass tolerance of ± 1 Da.

Consecutive sections were used for immunohistochemical stainings to validate the Tβ-4 identification of Maier et al. with respect to the histological location [25]. Briefly, sections were rehydrated in PBS, blocked with 10% goat serum for 30 min (Dako, Denmark), and incubated with primary antibodies against Tβ-4 (TMSB4X purified MaxPab mouse antibody, diluted 1:100, Abnova) overnight at 4°C. Staining was performed using the Streptavidin/HRP kit (Dako, Denmark) and the DAB chromogen system (Dako, Denmark) according to the manufacturer's instructions. After gently rinsing with distilled water, slides were counterstained with hematoxylin (Roth, Germany).

Based on ploidy status assessment of 405 colorectal cancer specimens, tissues of nine diploid and nine aneuploid colorectal cancers as well as ten corresponding adjacent normal mucosa specimens from the same patients were selected. Additionally, all diploid and aneuploid colorectal cancer samples were equally subdivided into carcinomas with lymph node positive and negative metastasis, into UICC I/II and UICC III/IV cancers, and into patients with a survival of less and more than 60 months (Table 1b). Tissues were implemented into a tissue microarray in duplicates by using a semiautomated arrayer (TMArrayer, Pathology Devices, MD, USA) as described [8]. After deparaffinization, sections were incubated with the primary antibody against Tβ-4 (TMSB4X purified MaxPab mouse antibody, diluted 1:100, Abnova) overnight at 4°C. Staining was performed by means of the Streptavidin/HRP kit (Dako, Denmark) and the DAB chromogen system (Dako, Denmark) according to the manufacturer's instructions.

For all samples, immunopositivity of Tβ-4 staining in epithelial cells was analyzed using ImageScope™ software version 11.2.0.780 with the supplied positive pixel count algorithm v9.1 (Aperio Technologies, CA, USA). ImageScope™'s basic function is to view digital slides created by a microscope slide scanner from glass tissue slides and to evaluate selected regions using provided and purchased algorithms. When available, positivity was averaged over sample duplicates. For survival analysis, the TMA stain was scored semiquantitatively based on the best sensitivity and specificity between diploid and aneuploid colon cancers: weakly staining (positivity count <0.4489) and strong staining (positivity count >0.4489).

### Statistical analyses

On the basis of the anatomical/pathological classification, regions of interest (ROIs) were defined in every tissue section using the FlexImaging 2.1 software (Bruker Daltonic GmbH, Germany) in order to select spectra associated to the groups to be analyzed. In particular, three classes of spectra were created (aneuploid carcinoma, diploid carcinoma and normal mucosa) and two comparisons were performed: normal mucosa vs. carcinoma (aneuploid plus diploid) and diploid vs. aneuploid carcinoma. Statistical analyses were carried out using the ClinProTools 2.2 software (Bruker Daltonic GmbH, Germany). These extracted mass spectra underwent recalibration by spectral alignment, normalization based on their total ion count in the observation mass range, and baseline correction using the Convex Hull algorithm with baseline flatness of 0.8, 1,000 ppm maximum peak shift, and exclusion of null spectra. An average spectrum created for each class was used for peak picking and to define integration ranges for obtaining intensities or areas of each peak in every single spectrum. Peak areas were then used for calculation, using the end-point level integration type. In order to generate a model useful to discriminate peaks belonging to the compared classes, three different algorithms were tested: quick classifier, genetic algorithm and supervised neural network. The model with the higher recognition capability was then used to classify new, independent ROIs and whole tissue slides. Exporting the results obtained into FlexImaging enabled us to visualize the classification in a colour-encoded depiction. Additionally, a receiver operator characteristic (ROC) analysis was performed in order to highlight peaks which better discriminate [area under curve (AUC) >0.75] among the compared classes. These peaks were finally compared to those chosen by the selected algorithm. Regarding immunohistochemistry, Mann-Whitney U test were used to test the observed Tβ-4 immunopositivity differences. Duplicated TMA-cores per case were averaged. Kaplan-Meier curves were calculated and tested for significant differences by the logrank test.

## SUPPLEMENTAL DATA


