# Supplementary material for: MALDI-imaging reveals thymosin beta-4 as an independent prognostic marker for colorectal cancer
Source: Oncotarget. 2015 Nov 5;6(41):43869–80. doi: 10.18632/oncotarget.6103 (PMC4791273; doi:10.18632/oncotarget.6103)
Supplement: Supplementary file 1 [file oncotarget-06-43869-s001.pdf]

SUPPLEMENTAL DATA

Sektion für Translationale Chirurgische Onkologie & Biomaterialbanken
Universität zu Lübeck
Ratzeburger Allee 160
23562 Lübeck

Material: Nr: Eing.-Nr.: 07.09.15

Diagnose: Name: T1

Geb.Dat.:

Cytometrische DNS-Analyse AHRENS ICM Cytometrie-System

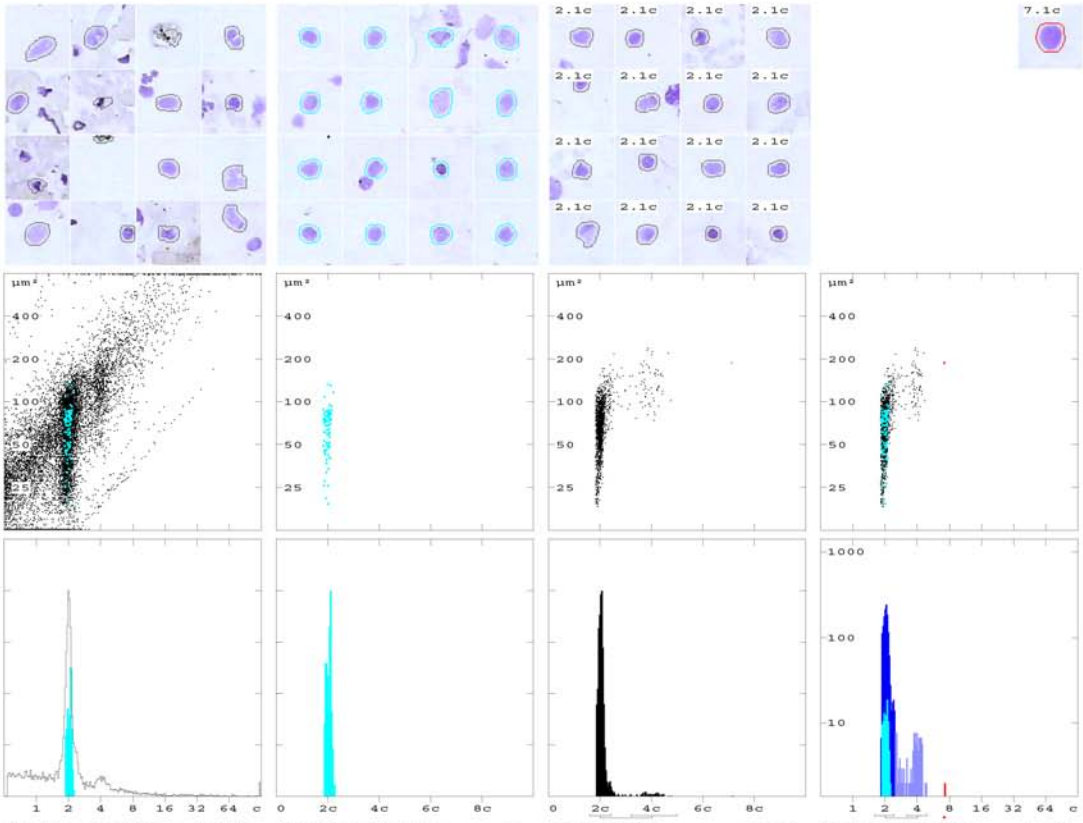

detekt. Partikel: 11801
Messdatum: 07.09.15
20x Nikon Eclipse Ci-S
PXC JAI
BL:23 Glare-Korr: 2%

Referenz-Zellen: 88
Variationskoeff. 4.6%
Korrel.IOD/Fläche 0.25
IOD: 12.22
mittl.Fläche 67µm²

Stammlinie: 2.02c
gesamt 1590
Variationskoeff. 4.8%
S 50 3.1%
G2/M 44 2.8%
G2/M-Exc 1 0.1%

Analyse-Zellen: 1598
Ploidie 1.8c - 7.1c
5c-Exc 1
9c-Exc 0
SSI 8.0%

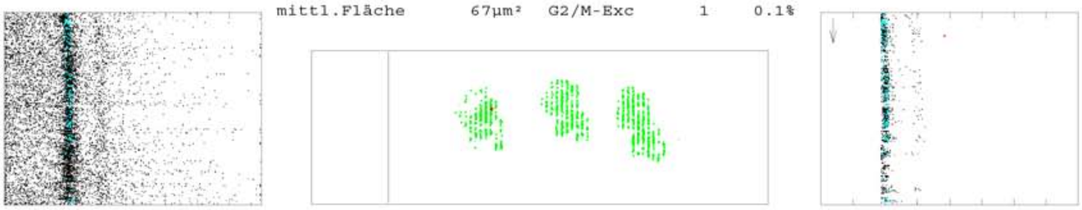

Grenzfall: eine (aneuploide) Einzelzelle oberhalb 5c (bei 7.1c)
diploide Stammlinie mit leichter Proliferation

(continued)

Sektion für Translationale Chirurgische Onkologie & Biomaterialbanken
Universität zu Lübeck
Ratzeburger Allee 160
23562 Lübeck

Material: Nr: Eing.-Nr.: 07.09.15

Diagnose: Name: T2

Geb.Dat.:

Cytometrische DNS-Analyse AHRENS ICM Cytometrie-System

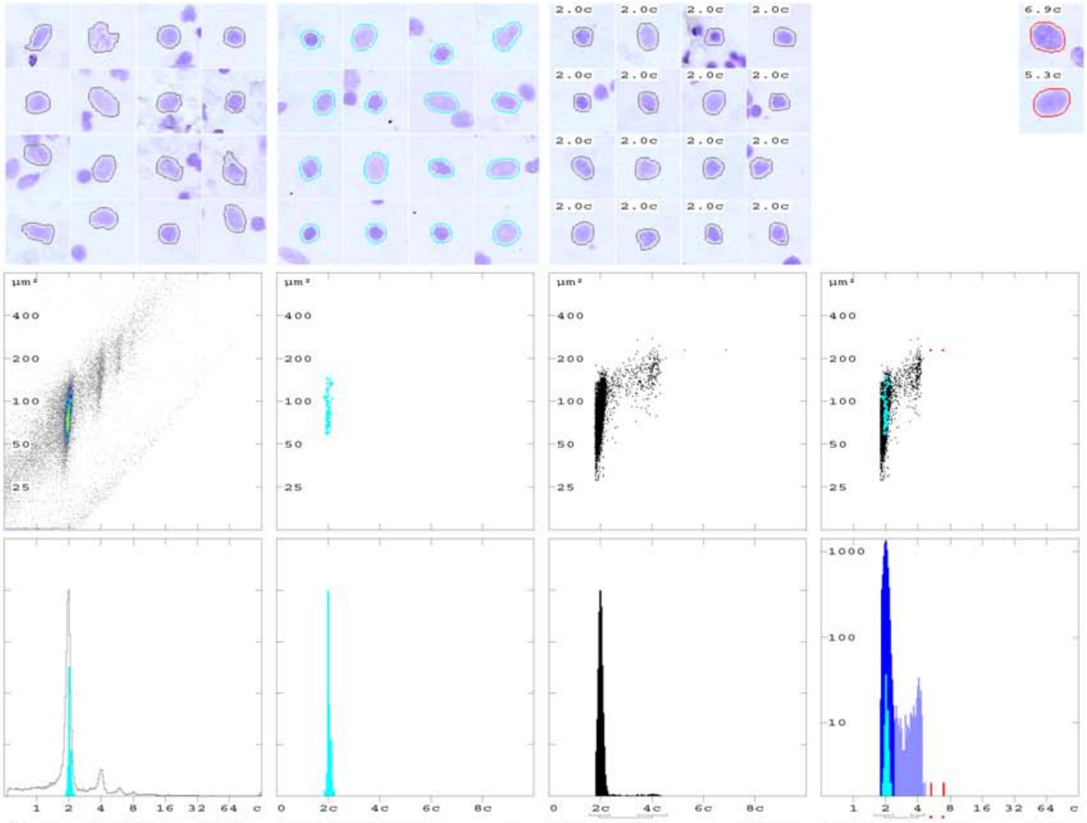

detekt. Partikel: 25586
Messdatum: 07.09.15
20x Nikon Eclipse Ci-S
PXC JAI
BL:23 Glare-Korr: 2%

Referenz-Zellen: 81
Variationskoeff. 3.0%
Korrel.IOD/Fläche 0.18
IOD: 12.95
mittl.Fläche 91µm²

Stammlinie: 1.96c
gesamt 8214
Variationskoeff. 4.7%
S 144 1.8%
G2/M 162 2.0%
G2/M-Exc 2 0.0%

Analyse-Zellen: 8284
Ploidie 1.8c - 6.9c
5c-Exc 2
9c-Exc 0
SSI 6.5%

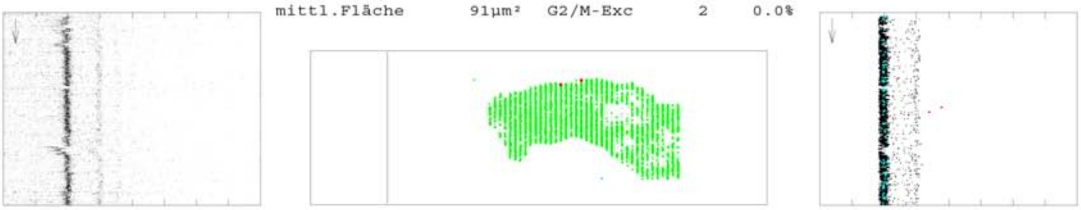

2 (aneuploide) Einzelzellen oberhalb 5c (bis 6.9c)
diploide Stammlinie mit leichter Proliferation

(*continued*)

Sektion für Translationale Chirurgische Onkologie & Biomaterialbanken
Universität zu Lübeck
Ratzeburger Allee 160
23562 Lübeck

Material:	Nr:	Eing.-Nr.:	07.09.15
Diagnose:		Name: T3	
		Geb.Dat.:	

Cytometrische DNS-Analyse AHRENS ICM Cytometrie-System

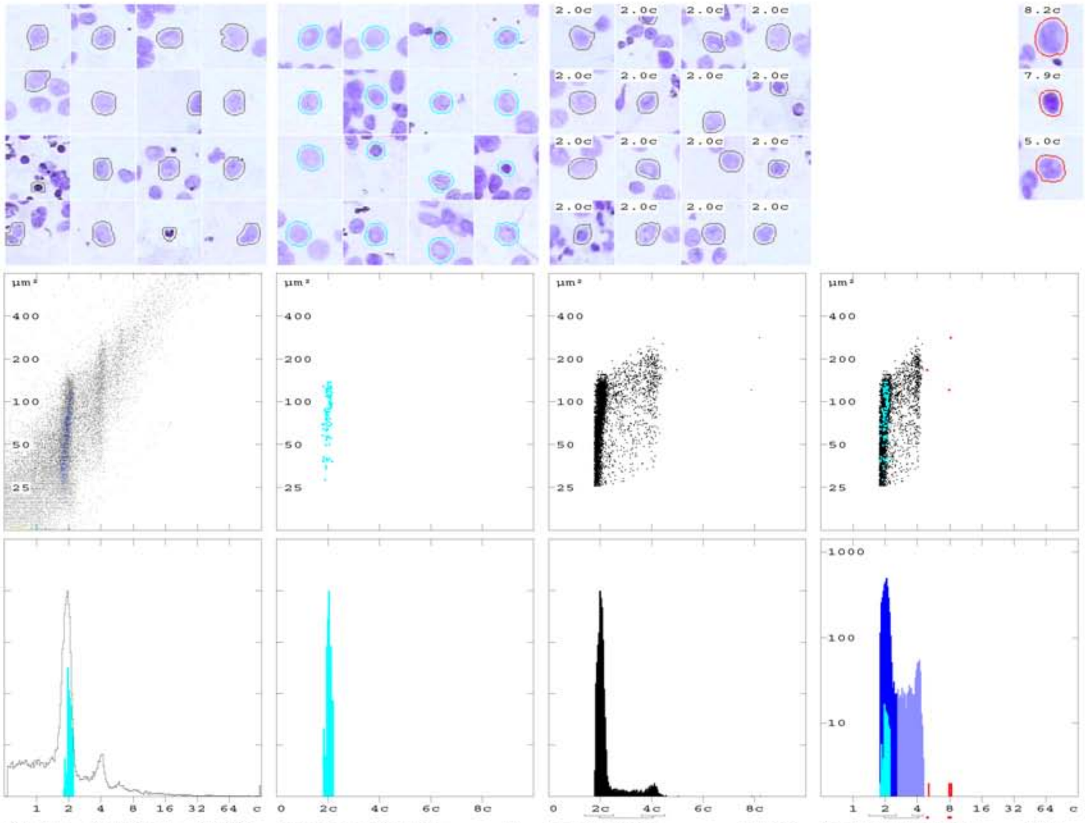

detekt. Partikel:	29303	Referenz-Zellen:	84	Stammlinie:	1.97c	Analyse-Zellen:	4791
Messdatum:	07.09.15			gesamt	4737	Ploidie	1.8c - 8.2c
20x Nikon Eclipse Ci-S		Variationskoeff.	4.7%	Variationskoeff.	6.2%	5c-Exc	2
PXC JAI		Korrel.IOD/Fläche	0.36	S 597	12.6%	9c-Exc	0
BL:23	Glare-Korr: 2%	IOD:	12.47	G2/M 226	4.8%	SSI	18.8%
		mittl.Fläche	84µm²	G2/M-Exc 3	0.1%		

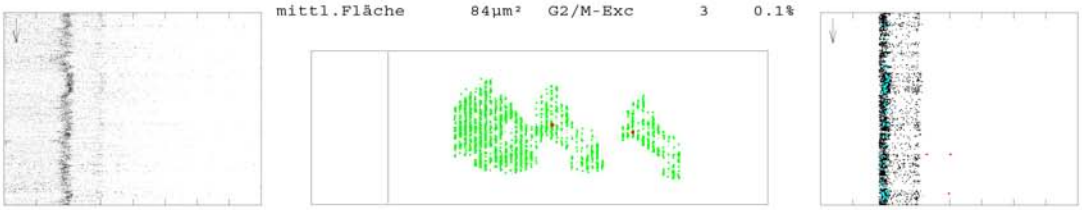

2 (aneuploide) Einzelzellen oberhalb 5c (bis 8.2c)
diploide Stammlinie mit erhöhter Proliferation

(*continued*)

Sektion für Translationale Chirurgische Onkologie & Biomaterialbanken
Universität zu Lübeck
Ratzeburger Allee 160
23562 Lübeck

Material: Nr: Eing.-Nr.: 07.09.15

Diagnose: Name: T4

Geb.Dat.: 01.01.1900

Cytometrische DNS-Analyse AHRENS ICM Cytometrie-System

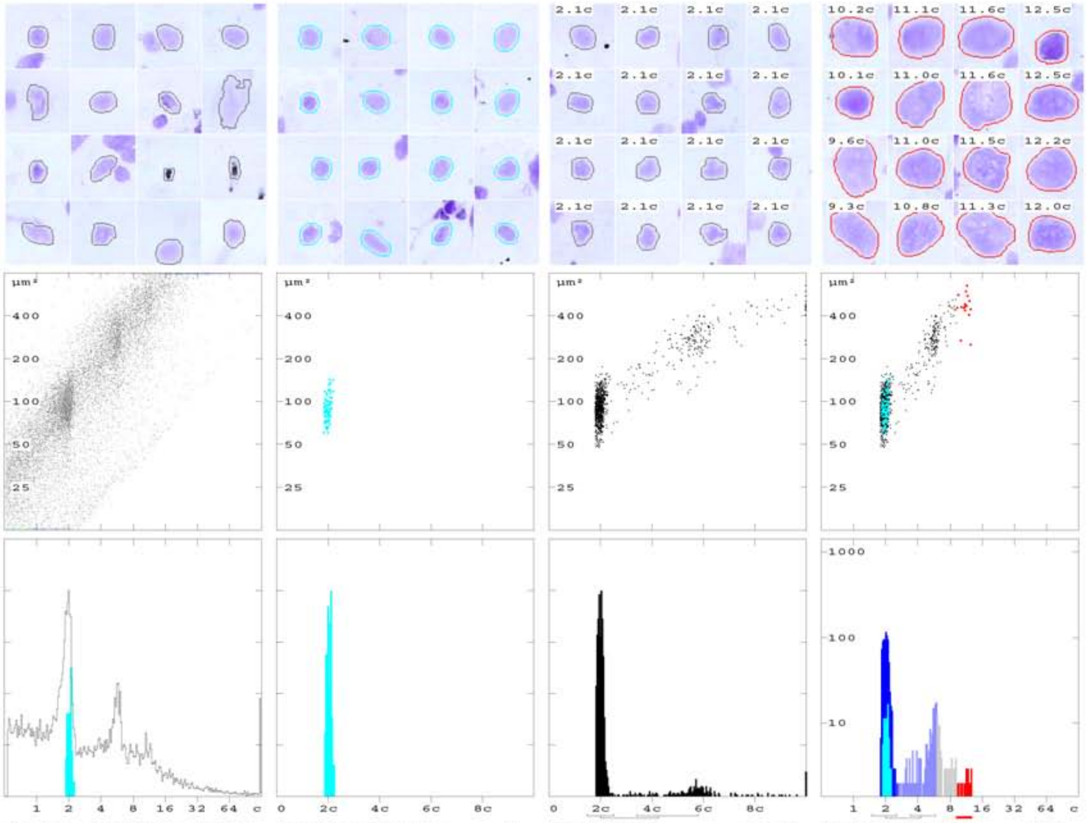

detekt. Partikel: 19765
Messdatum: 06.09.13
20x Nikon Eclipse Ci-S
PXC JAI
BL:23 Glare-Korr: 5%

Referenz-Zellen: 74
Variationskoeff. 4.4%
Korrel.IOD/Fläche 0.39
IOD: 12.29
mittl.Fläche 91µm²

Stammlinie: 2.01c
gesamt 990
Variationskoeff. 5.9%
S 33 3.3%
G2/M 102 10.3%
G2/M-Exc 91 9.2%

Analyse-Zellen: 1083
Ploidie 1.8c - 12.5c
5c-Exc 162
9c-Exc 18
SSI 18.4%

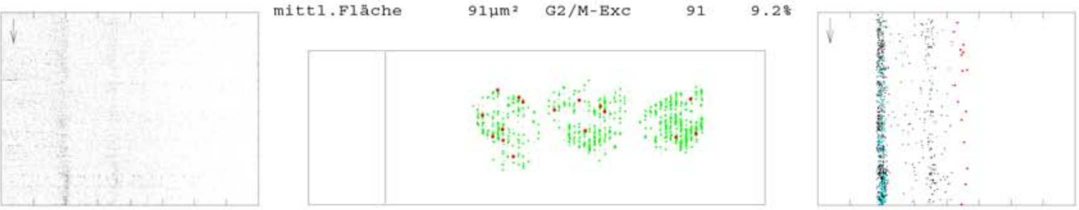

162 (aneuploide) Einzelzellen oberhalb 5c und 10c (bis 12.5c)
diploide Stammlinie mit leichter Proliferation

(*continued*)

Sektion für Translationale Chirurgische Onkologie & Biomaterialbanken
Universität zu Lübeck
Ratzeburger Allee 160
23562 Lübeck

Material:	Nr:	Eing.-Nr.:	01.01.02
Diagnose:		Name:	T5
		Geb.Dat.:	01.01.1900

Cytometrische DNS-Analyse AHRENS ICM Cytometrie-System

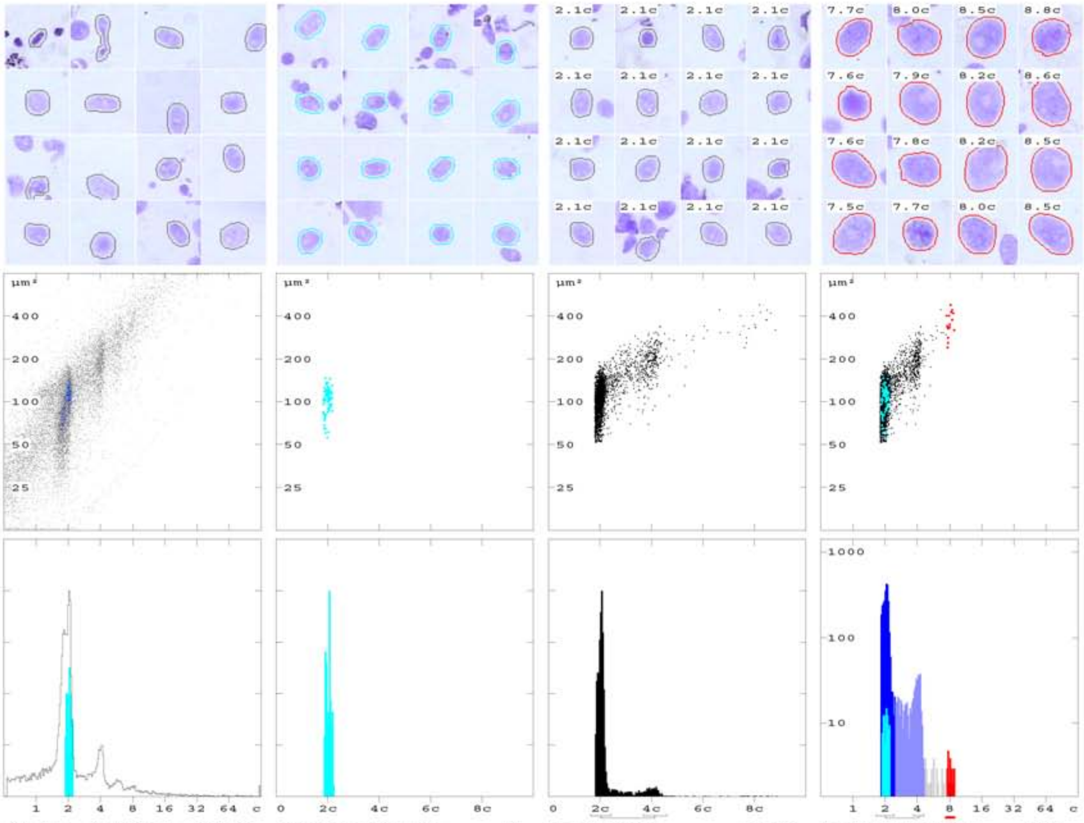

detekt. Partikel: 19083	Referenz-Zellen: 80	Stammlinie: 2.03c	Analyse-Zellen: 3621
Messdatum: 01.01.02		gesamt 3468	Ploidie 1.8c - 8.8c
20x Nikon Eclipse Ci-S	Variationskoeff. 4.9%	Variationskoeff. 4.4%	5c-Exc 30
PXC JAI	Korrel.IOD/Fläche 0.25	S 283 8.2%	9c-Exc 0
BL:23 Glare-Korr: 2%	IOD: 13.12	G2/M 199 5.7%	SSI 13.6%
	mittl.Fläche 103µm²	G2/M-Exc 36 1.0%	

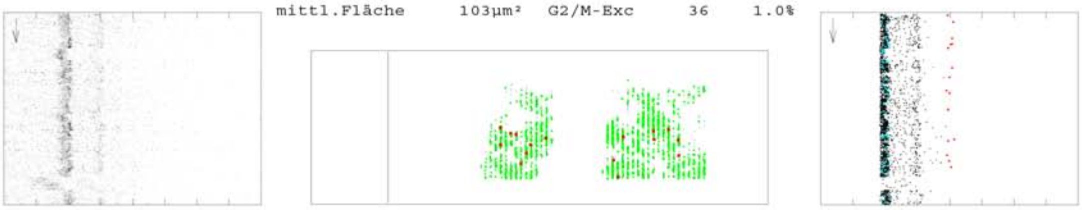

30 (aneuploide) Einzelzellen oberhalb 5c (bis 8.8c)
diploide Stammlinie mit erhöhter Proliferation

(*continued*)

Sektion für Translationale Chirurgische Onkologie & Biomaterialbanken
Universität zu Lübeck
Ratzeburger Allee 160
23562 Lübeck

Material: Nr: Eing.-Nr.: 07.09.15

Diagnose: Name: T6

Geb.Dat.: 01.01.1900

Cytometrische DNS-Analyse AHRENS ICM Cytometrie-System

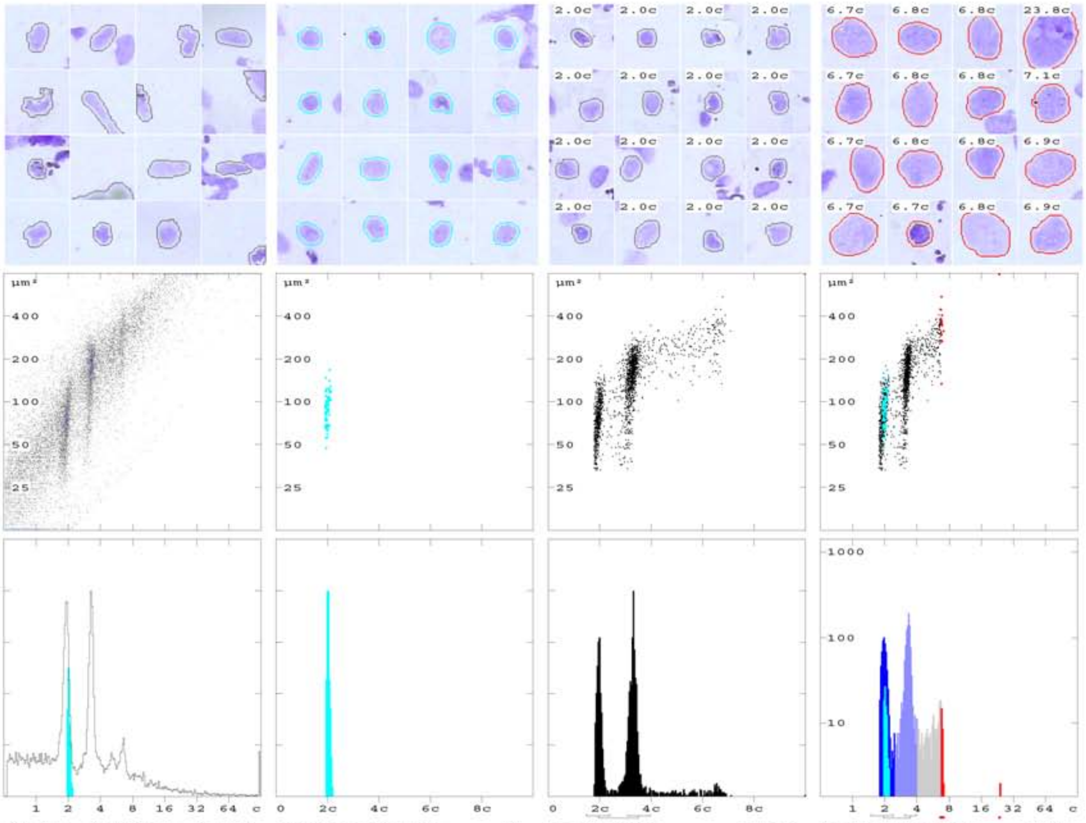

detekt. Partikel: 21468
Messdatum: 01.01.02
20x Nikon Eclipse Ci-S
PXC JAI
BL:23 Glare-Korr: 2%

Referenz-Zellen: 80
Variationskoeff. 2.8%
Korrel.IOD/Fläche 0.39
IOD: 12.92
mittl.Fläche 90µm²

Stammlinie: 1.95c
gesamt 1591
Variationskoeff. 5.2%
S 101 6.3%
G2/M 891 56.0%
G2/M-Exc 203 12.8%

Analyse-Zellen: 1912
Ploidie 1.8c - 23.8c
5c-Exc 142
9c-Exc 1
SSI 24.3%

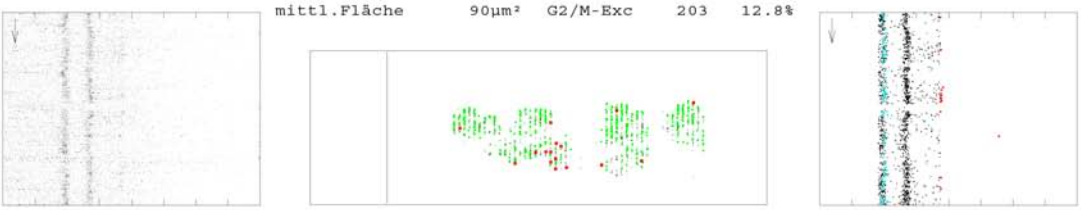

142 (aneuploide) Einzelzellen oberhalb 5c und 10c (bis 23.8c)
diploide Stammlinie mit erhöhter Proliferation

Supplemental Data S1:

(*continued*)

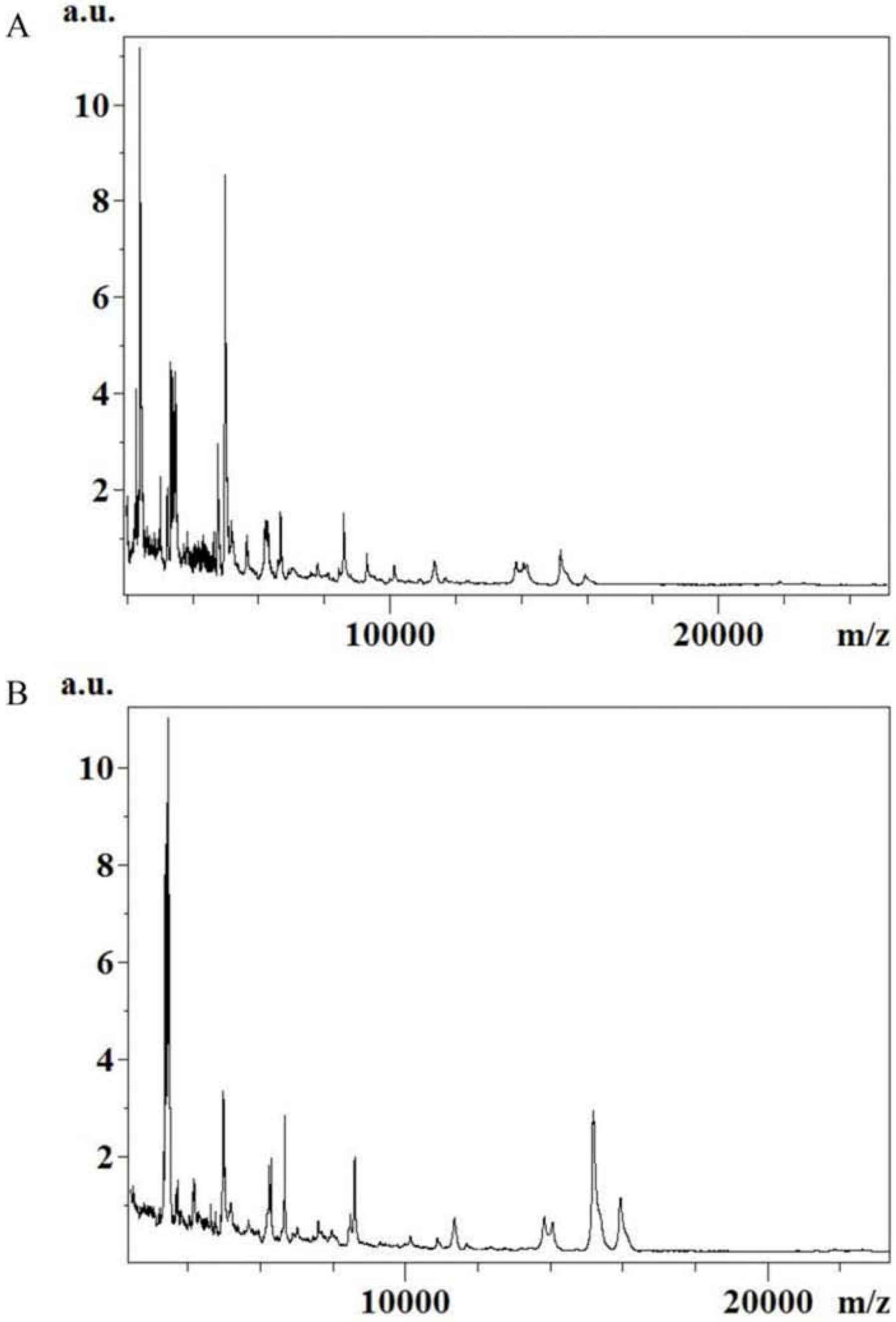

(*continued*)

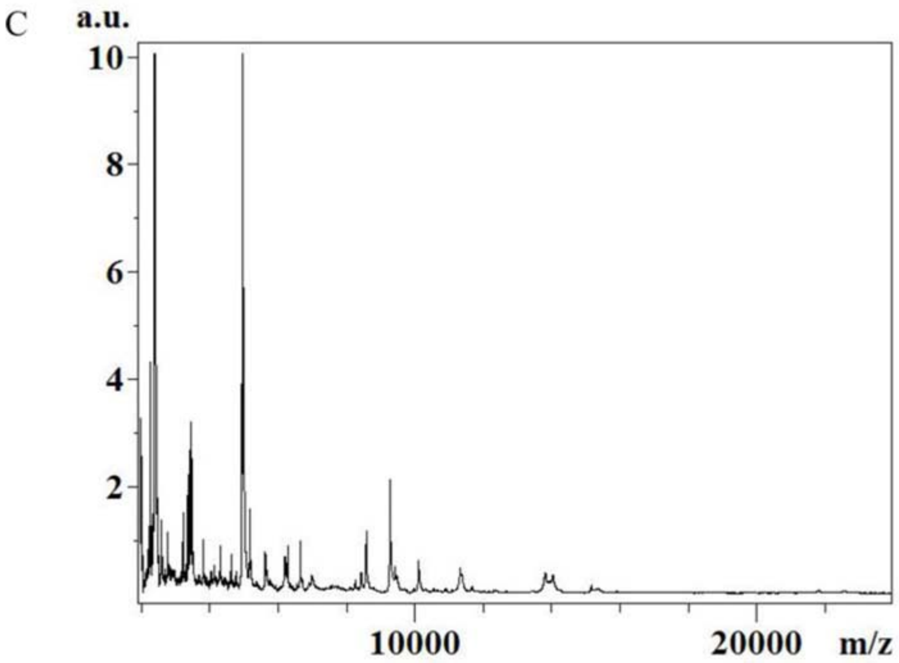

Supplemental Data S2 (*Continued*): Average protein spectra of one normal mucosa A. as well as one diploid B. and one aneuploid. C. colorectal cancer sample. Data were obtained with FlexImaging software.

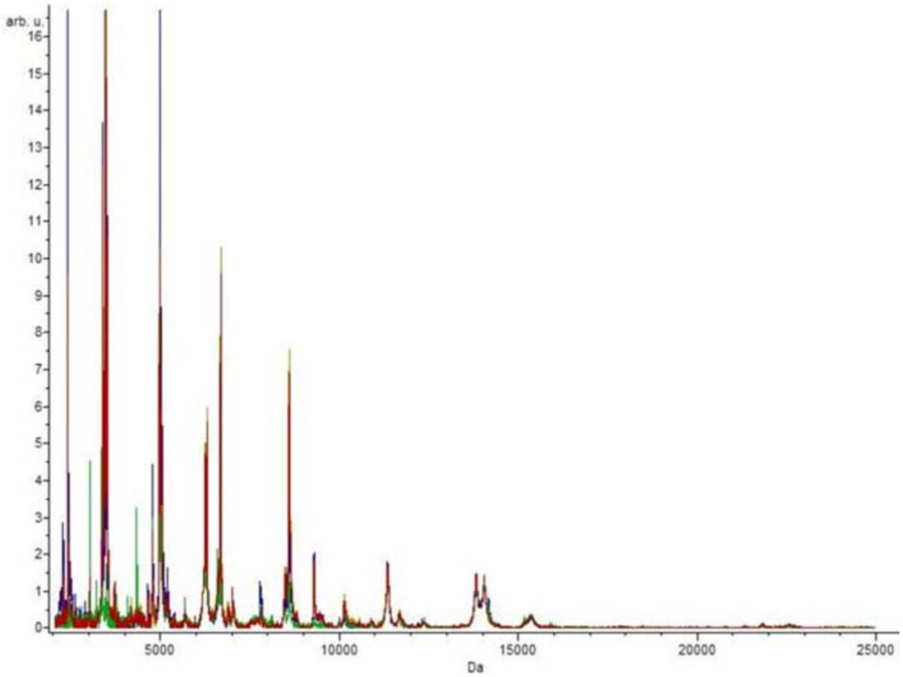

Supplemental Data S3: Overall sum spectra of normal mucosa (green), colorectal carcinoma (red) as well as one diploid (yellow) and one aneuploid (blue) colorectal cancer sample. Data were obtained with ClinProTools software.

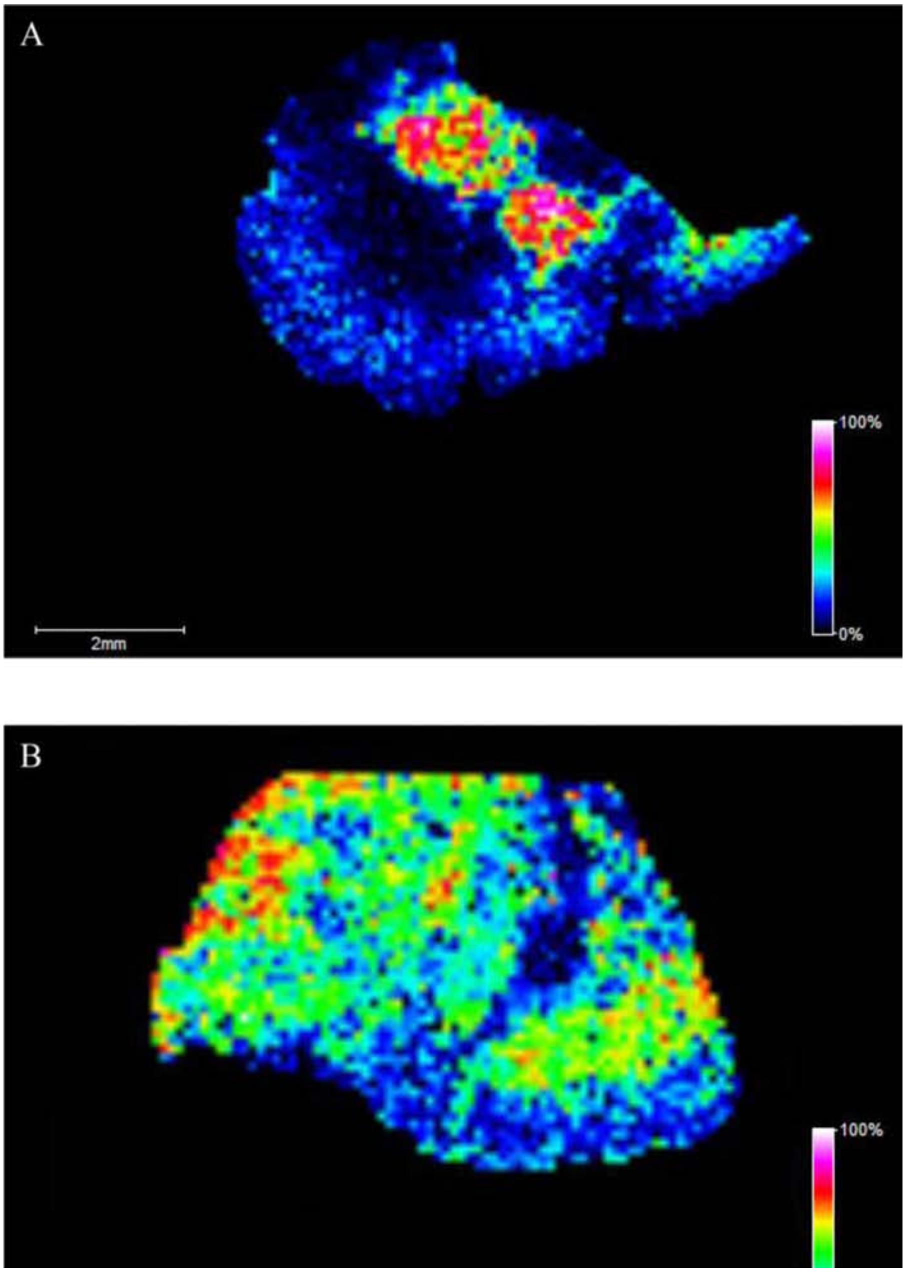

(*continued*)

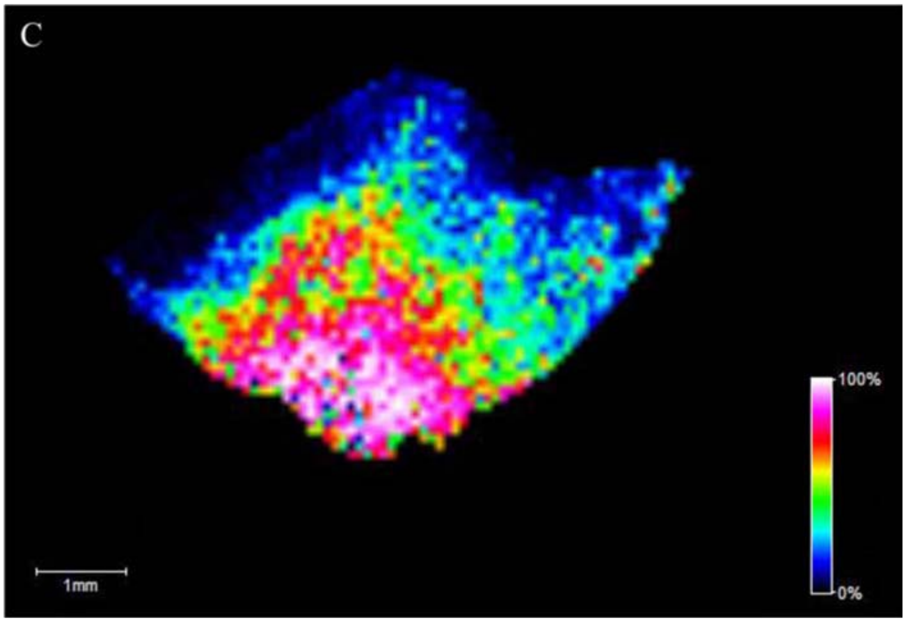

Supplemental Data S4 (*Continued*): Average ion intensity spectra of Tβ-4 (m/z 4,977) in normal mucosa (sample T6), A. as well as diploid (sample T1), B. and aneuploid (sample N6), C. colorectal cancers. Data were obtained with ClinProTools software.

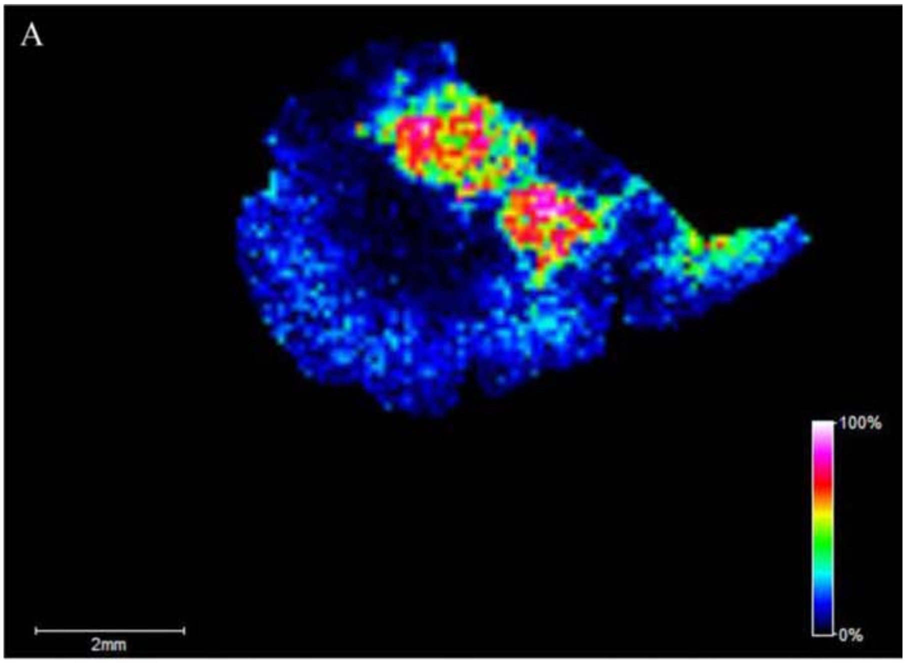

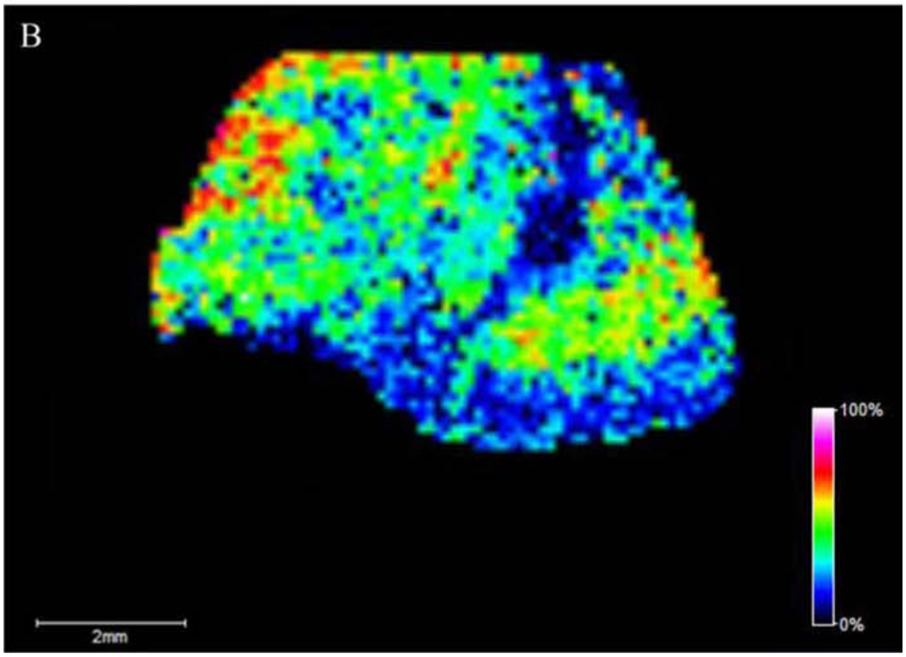

(*continued*)

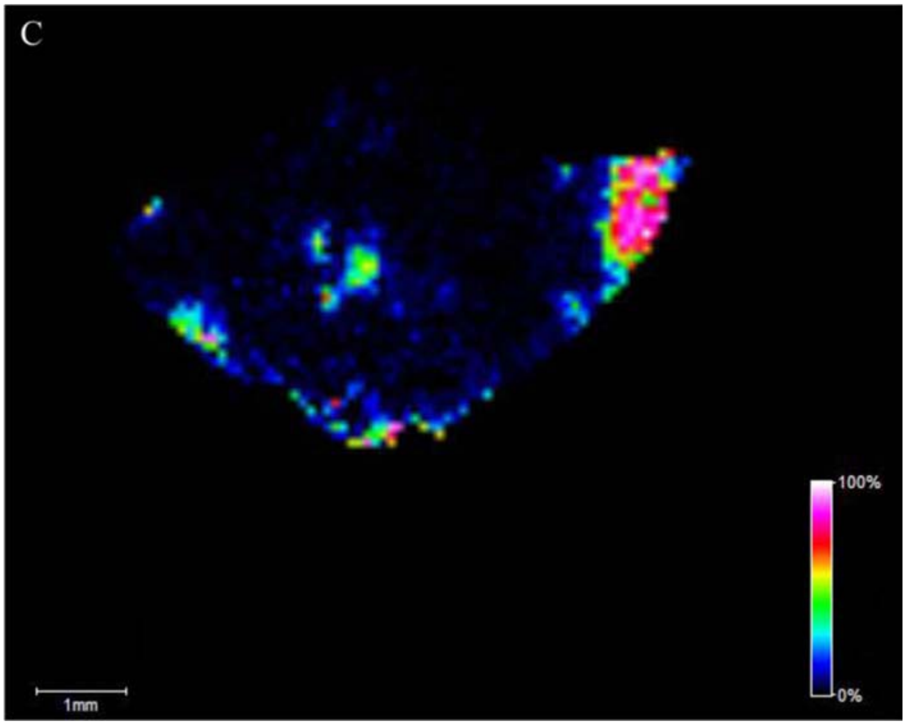

Supplemental Data S5 (*Continued*): Average ion intensity spectra of 14–3-3 protein sigma (m/z 3,376) in normal mucosa (sample T6), A. as well as diploid (sample T1), B. and aneuploid (sample N6), C. colorectal cancers. Data were obtained with ClinProTools software.

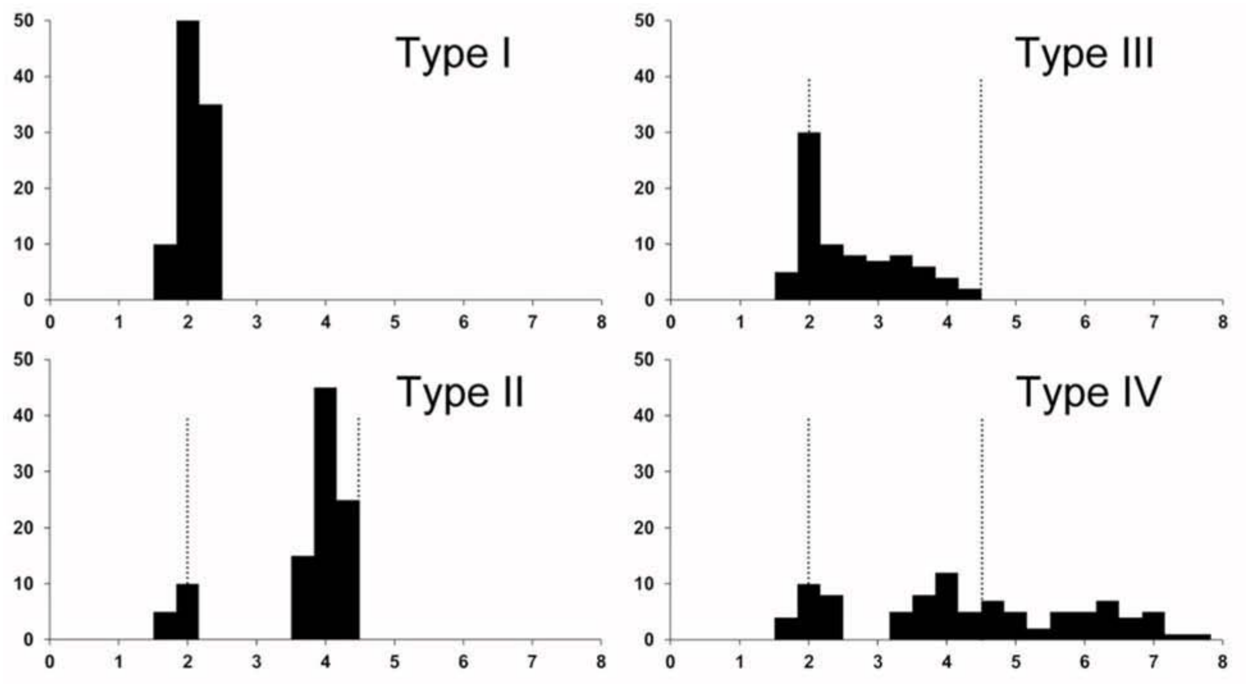

Supplemental Data S6: DNA histogram types according to the Auer classification. While DNA histograms of types I, II, and III characterize euploid cell populations, type IV histograms reflect aneuploid populations with decreased genomic stability. Displayed are number of cells (*y*-axis) and DNA content (*x*-axis).
